# Supplementary material for: Survival cost sharing among altruistic full siblings in Mendelian population
Source: BMC Ecol Evol. 2024 Nov 18;24:142. doi: 10.1186/s12862-024-02317-z (PMC11572324; doi:10.1186/s12862-024-02317-z)
Supplement: Supplementary file 1 — Supplementary Material 1. [file 12862_2024_2317_MOESM1_ESM.docx]

Supporting Information

**Survival cost sharing among altruistic full siblings in Mendelian population**

József Garay, Inmaculada López, Zoltán Varga, Villő Csiszár, Tamás F. Móri

Since this is a purely theoretical work, we do not deal with data. As for the method, our study is built upon the concept of evolutionary stability, adapted to a population model based on the general mating table. Our results were obtained using mathematical analysis and differential equations. The numerical simulations were carried out in MATLAB R2023a environment. The work can be repeated following the calculations of the SI.

**SI-A: Static condition for evolutionarily stability**

**Phenotypic group determines payoff.** Each sibling may have one of two phenotypes, altruistic and selfish, and this phenotype is genetically fixed. Homozygotes $G_{1}=\left( \left[ a \right],\left[ a \right] \right)$and $G_{3}=\left( \left[ A \right],\left[ A \right] \right)$ are altruist and selfish, resp., and heterozygote $G_{2}=\left( \left[ a \right],\left[ A \right] \right)$ is either altruistic or selfish. We will analyze the following general setup. The phenotype of a heterozygote individual is altruistic with probability $p\in\left[ 0, 1 \right]$, and selfish with the complementary probability $1-p$. Particularly, altruist is recessive, dominant, or additive, according that $p=0, 1,$or $\frac{1}{2}$. In our $n$-person donation game there are exactly $n$ siblings in each family.

**Cost sharing.** Each sibling having at least one altruistic sib receives benefit $b$ altogether, which is collected from its altruistic sibs equally. Thus, in a family with $k$ altruistic and $n-k$ selfish siblings the payoff of each altruist is equal to $a-\left( n-1 \right)c$, if $k=1$, and to $a+b-\left( k-1 \right)\frac{c}{k-1}-\left( n-k \right)\frac{c}{k}=a+b-\frac{n}{k}c$, if $k\geq2$. At the same time, each selfish individual’s payoff is $a+b$, if $k\geq1$, and $a$, if $k=0$. For the sake of simplicity, $c\leq a/\left( n-1 \right)$ and $b\leq1-a$ is assumed.

Let $n_{i(jk)}$ denote the expected number of (geno)type $i$ offspring in a family of type $G_{j}\times G_{k}$, $i,j,k\in\left\{ 1,2,3 \right\}$. In what follows we compute these quantities. Throughout, $\mathbb{I}\left( . \right)$ will denote the indicator of the random event in brackets.

Let $X_{cc}\boldsymbol{,} X_{hc}, X_{hd}, X_{dd}$ denote the (random) number of homozygote altruistic, heterozygote altruistic, heterozygote selfish, and homozygote selfish offspring, resp., in the family under consideration. Their joint distribution is multinomial (permitting degenerate ones with one or more parameters being zero).

For each family type,

$$n_{1\left( ij \right)}=\left( a-\left( n-1 \right)c \right)\mathbb{P}\left( X_{cc}=1,X_{hc}=0 \right)\mathbb{+E}\left( X_{cc}\left( a+b-\frac{n}{X_{cc}+X_{hc}}c \right)\mathbb{I}\left( X_{cc}+X_{hc}\geq2 \right) \right),$$

$$n_{2\left( ij \right)}=\left( a-\left( n-1 \right)c \right)\mathbb{P}\left( X_{cc}=0,X_{hc}=1 \right)\mathbb{+E}\left( X_{hc}\left( a+b-\frac{n}{X_{cc}+X_{hc}}c \right)\mathbb{I}\left( X_{cc}+X_{hc}\geq2 \right) \right)\mathbb{+E}\left( X_{hd}\left( a+b \mathbb{I}\left( X_{cc}+X_{hc}\geq1 \right) \right) \right),$$

$$n_{3(ij)}\mathbb{=E}\left( X_{dd}\left( a+b\mathbb{I}\left( X_{cc}+X_{hc}\geq1 \right) \right) \right).$$

Suppose the joint distribution of $\left( X_{cc}\boldsymbol{,} X_{hc}, X_{hd}, X_{dd} \right)$ is general multinomial $M\left( n, r_{1}, r_{2}, r_{3}, r_{4} \right)$. Then

$$\mathbb{P}\left( X_{cc}=1, X_{hc}=0 \right)=nr_{1}\left( r_{3}+r_{4} \right)^{n-1},$$

and the conditional distribution of $X_{cc}$ given $X_{cc}+X_{hc}=k$ is binomial $B\left( k, \frac{r_{1}}{r_{1}+r_{2}} \right)$, hence

$$\mathbb{E}\left( X_{cc}\left( a+b-\frac{n}{X_{cc}+X_{hc}}c \right) | X_{cc}+X_{hc}=k \right)=k\frac{r_{1}}{r_{1}+r_{2}}\left( a+b-\frac{n}{k}c \right)=\frac{r_{1}}{r_{1}+r_{2}}\left( k\left( a+b \right)-nc \right).$$

The distribution of $X_{cc}+X_{hc}$ is binomial $B\left( n, r_{1}+r_{2} \right)$, thus

$$\mathbb{E}\left( \left( X_{cc}+X_{hc} \right)\mathbb{I}\left( X_{cc}+X_{hc}\geq2 \right) \right)= n\left( r_{1}+r_{2} \right)\left( 1 -\left( r_{3}+r_{4} \right)^{n-1} \right),$$

$$\mathbb{P}\left( X_{cc}+X_{hc}\geq2 \right)=1-\left( r_{3}+r_{4} \right)^{n-1}\left( r_{3}+r_{4}+n\left( r_{1}+r_{2} \right) \right)=1-\left( r_{3}+r_{4} \right)^{n-1}\left( 1+\left( n-1 \right)\left( r_{1}+r_{2} \right) \right).$$

From all these it follows that

$$\mathbb{E}\left( X_{cc}\left( a+b-\frac{n}{X_{cc}+X_{hc}}c \right)\mathbb{I}\left( X_{cc}+X_{hc}\geq2 \right) \right)\mathbb{=E}\left( \left( X_{cc}+X_{hc} \right)\mathbb{I}\left( X_{cc}+X_{hc}\geq2 \right) \right)\frac{r_{1}}{r_{1}+r_{2}}\left( a+b \right)\mathbb{-P}\left( X_{cc}+X_{hc}\geq2 \right)\frac{r_{1}}{r_{1}+r_{2}}nc=nr_{1}\left( 1 -\left( r_{3}+r_{4} \right)^{n-1} \right)\left( a+b \right)-\left( 1-\left( r_{3}+r_{4} \right)^{n-1}\left( 1+\left( n-1 \right)\left( r_{1}+r_{2} \right) \right) \right)\frac{r_{1}}{r_{1}+r_{2}}nc,$$

and consequently,

$$n_{1\left( ij \right)}=\left( a-\left( n-1 \right)c \right) nr_{1}\left( r_{3}+r_{4} \right)^{n-1}+nr_{1}\left( 1 -\left( r_{3}+r_{4} \right)^{n-1} \right)\left( a+b \right)-\left( 1-\left( r_{3}+r_{4} \right)^{n-1} \right)\frac{r_{1}}{r_{1}+r_{2}}nc+\left( r_{3}+r_{4} \right)^{n-1}\left( n-1 \right)nr_{1}c=nr_{1}a+nr_{1}\left( 1 -\left( r_{3}+r_{4} \right)^{n-1} \right)b-\left( 1-\left( r_{3}+r_{4} \right)^{n-1} \right)\frac{1}{r_{1}+r_{2}}nr_{1}c.$$

Let us turn to $n_{2\left( ij \right)}$. As we have seen above,

$$n_{2\left( ij \right)}=\left( a-\left( n-1 \right)c \right)\mathbb{P}\left( X_{cc}=0,X_{hc}=1 \right)\mathbb{+E}\left( X_{hc}\left( a+b-\frac{n}{X_{cc}+X_{hc}}c \right)\mathbb{I}\left( X_{cc}+X_{hc}\geq2 \right) \right)\mathbb{+E}\left( X_{hd}\left( a+b \mathbb{I}\left( X_{cc}+X_{hc}\geq1 \right) \right) \right).$$

By analogy, the sum of the first two terms in the right-hand side is equal to

$$nr_{2}a+nr_{2}\left( 1 -\left( r_{3}+r_{4} \right)^{n-1} \right)b-\left( 1-\left( r_{3}+r_{4} \right)^{n-1} \right)\frac{1}{r_{1}+r_{2}}nr_{2}c.$$

The last term can be treated as follows.

$$\mathbb{E}\left( X_{hd}\left( a+b \mathbb{I}\left( X_{cc}+X_{hc}\geq1 \right) \right) \right)\mathbb{=E}\left( X_{hd} \right)\left( a+b \right)\mathbb{-E}\left( X_{hd}\mathbb{I}\left( X_{cc}=X_{hc}=0 \right) \right)b.$$

Here $X_{hd}$ is binomial $B\left( n, r_{3} \right)$, hence $\mathbb{E}\left( X_{hd} \right)=nr_{3}$. Moreover,

$$\mathbb{E}\left( X_{hd}\mathbb{I}\left( X_{cc}=X_{hc}=0 \right) \right)=\sum_{k=0}^{n} k\binom{n}{k}r_{3}^{k}r_{4}^{n-k}=nr_{3}\left( r_{3}+r_{4} \right)^{n-1},$$

thus

$$\mathbb{E}\left( X_{hd}\left( a+b \mathbb{I}\left( X_{cc}+X_{hc}\geq1 \right) \right) \right)= nr_{3}a+nr_{3}\left( 1-\left( r_{3}+r_{4} \right)^{n-1} \right)b.$$

Consequently,

$$n_{2\left( ij \right)}=n\left( r_{2}+r_{3} \right)a+n\left( r_{2}+r_{3} \right)\left( 1 -\left( r_{3}+r_{4} \right)^{n-1} \right)b-\left( 1-\left( r_{3}+r_{4} \right)^{n-1} \right)\frac{1}{r_{1}+r_{2}}nr_{2}c.$$

Finally, $n_{3\left( ij \right)}$ can be obtained from the third term of $n_{2\left( ij \right)}$ by interchanging $r_{3}$ and $r_{4}$, thus

$$n_{3\left( ij \right)}= nr_{4}a+nr_{4}\left( 1-\left( r_{3}+r_{4} \right)^{n-1} \right)b.$$

Let us specialize our results for each possible family types. In each cases we only have to find the probability distribution $\left( r_{1}, r_{2}, r_{3}, r_{4} \right)$, and plug it into the formulas above.

**Family type** $G_{1}\times G_{1}$. Then $r_{1}=1, r_{2}= r_{3}=r_{4}=0$, thus

$$n_{1(11)}=n\left( a+b-c \right), n_{2(11)}=n_{3(11)}=0.$$

**Family type** $G_{1}\times G_{2}$. Then $r_{1}=\frac{1}{2}, r_{2}=\frac{p}{2}, r_{3}=\frac{1-p}{2}, r_{4}=0$. Consequently,

$$n_{1\left( 12 \right)}=\frac{n}{2}a+\frac{n}{2}\left( 1 -\left( \frac{1-p}{2} \right)^{n-1} \right)b-\frac{n}{1+p}\left( 1-\left( \frac{1-p}{2} \right)^{n-1} \right)c,$$

$$n_{2\left( 12 \right)}=\frac{n}{2}a+\frac{n}{2}\left( 1 -\left( \frac{1-p}{2} \right)^{n-1} \right)b-\frac{pn}{1+p}\left( 1-\left( \frac{1-p}{2} \right)^{n-1} \right)c,$$

$$n_{3\left( 12 \right)}=0.$$

**Family type** $G_{1}\times G_{3}$. Then $r_{1}=0, r_{2}=p, r_{3}=1-p, r_{4}=0$. Hence

$$n_{1\left( 13 \right)}=n_{3\left( 13 \right)}=0,$$

$$n_{2\left( 13 \right)}= na+n\left( 1 -\left( 1-p \right)^{n-1} \right)\left( b-c \right).$$

**Family type** $G_{2}\times G_{2}$. Then $r_{1}=\frac{1}{4}, r_{2}=\frac{p}{2}, r_{3}=\frac{1-p}{2}, r_{4}=\frac{1}{4} ,$ therefore

$$n_{1\left( 22 \right)}=\frac{n}{4}a+\frac{n}{4}\left( 1 -\left( \frac{3-2p}{4} \right)^{n-1} \right)b-\frac{n}{1+2p}\left( 1-\left( \frac{3-2p}{4} \right)^{n-1} \right)c,$$

$$n_{2\left( 22 \right)}=\frac{n}{2}a+\frac{n}{2}\left( 1 -\left( \frac{3-2p}{4} \right)^{n-1} \right)b-\frac{2pn}{1+2p}\left( 1-\left( \frac{3-2p}{4} \right)^{n-1} \right)c,$$

$$n_{3\left( 22 \right)}= \frac{n}{4}a+\frac{n}{4}\left( 1-\left( \frac{3-2p}{4} \right)^{n-1} \right)b.$$

**Family type** $G_{2}\times G_{3}$. Then $r_{1}=0, r_{2}=\frac{p}{2}, r_{3}=\frac{1-p}{2}, r_{4}=\frac{1}{2}$. Thus, $n_{1\left( 23 \right)}=0$,

$$n_{2\left( 23 \right)}=\frac{n}{2}a+\frac{n}{2}\left( 1 -\left( \frac{2-p}{2} \right)^{n-1} \right)b-n\left( 1-\left( \frac{2-p}{2} \right)^{n-1} \right)c,$$

$$n_{3\left( 23 \right)}=\frac{n}{2}a+\frac{n}{2}\left( 1-\left( \frac{2-p}{2} \right)^{n-1} \right)b.$$

**Family type** $G_{3}\times G_{3}$. Then $r_{1}=r_{2}=r_{3}=0, r_{4}=1,$consequently

$$n_{1\left( 33 \right)}=n_{2\left( 33 \right)}=0, n_{3\left( 33 \right)}=na.$$

**When is genotype** $G_{1}$ **evolutionarily stable?**

Let the pure altruistic genotype $G_{1}$ be resident. According to [1], it is evolutionarily stable, if $n_{1\left( 11 \right)}>2n_{2\left( 12 \right)}$, that is,

$$n\left( a+b-c \right)>na+n\left( 1 -\left( \frac{1-p}{2} \right)^{n-1} \right)b-\frac{2pn}{1+p}\left( 1-\left( \frac{1-p}{2} \right)^{n-1} \right)c.$$

If $p<1$, this means that

$$b\boldsymbol{>}\left( \frac{1-p}{1+p}\left( \frac{2}{1-p} \right)^{n-1}+\frac{2p}{1+p} \right)c.$$

If $p=1$ (altruism is dominant), then $n_{1\left( 11 \right)}=2n_{2\left( 12 \right)}$ , therefore we need to check the second order conditions of the ESGD (Theorem 1, Garay et al., 2019). In our particular case, where there is only one primary and one secondary mutant phenotype ($G_{2}$ and $G_{3}$, resp.), the general condition puts on the following simpler form: genotype $G_{1}$ is evolutionarily stable, if

$$\frac{n_{3\left( 22 \right)}}{n_{1\left( 11 \right)}}\cdot\min\left\{ 0, n_{1\left( 11 \right)}-2n_{2\left( 13 \right)} \right\}+{2n}_{1\left( 12 \right)}>n_{2\left( 22 \right)}+n_{3\left( 22 \right)}.$$

After some calculus we arrive at the condition

$$b>\frac{4^{n-1}+2}{3} c.$$

It is interesting that this condition coincides with that of the additive $\left( p=1/2 \right)$ case. In the recessive case the sufficient condition for altruism to be evolutionarily stable is

$$b>2^{n-1}c.$$

Note that for $n=2$ the condition is simply $b>2c$, independently of $p$, i.e., the classical Hamilton’s rule is valid. This is so because for $n=2$ our cost sharing model coincides with the classical model of altruistic actions without cost sharing.

**When is genotype** $\boldsymbol{G}_{\boldsymbol{3}}$ **evolutionarily stable and when is instable?**

By symmetry, conditions for the evolutionary stability of $G_{3}$ can be obtained from those of $G_{1}$ by interchanging 1 and 3 in the subscripts. Thus the primary condition is $n_{3\left( 33 \right)}>2n_{2\left( 23 \right)}$, that is,

$$na>2\left[ \frac{n}{2}a+\frac{n}{2}\left( 1 -\left( \frac{2-p}{2} \right)^{n-1} \right)b-n\left( 1-\left( \frac{2-p}{2} \right)^{n-1} \right)c \right],$$

which simply yields $2c>b$ whenever $p>0$. For $p=0$ we have $n_{3\left( 33 \right)}=2n_{2\left( 23 \right)}$, therefore the secondary condition

$$\frac{n_{1\left( 22 \right)}}{n_{3\left( 33 \right)}}\cdot\min\left\{ 0, n_{3\left( 33 \right)}-2n_{2\left( 13 \right)} \right\}+{2n}_{3\left( 23 \right)}>n_{2\left( 22 \right)}+n_{1\left( 22 \right)}$$

should be applied. After some calculus we get that this is also equivalent to $2c>b$.

Next, we are interested in the *instability* of $G_{3}$. Though it is true that reversing the inequality in the primary condition of stability is sufficient for instability, this is not necessarily so for the secondary condition. Thus all we can say is that the classical Hamilton’s rule $b>2c$ implies the instability of selfishness provided altruism is not recessive.

**SI-B: When is the net effect of the altruistic interaction remunerative for the altruist gene [a]?**

Firstly, let us compute the average benefit of the altruist gene [a] from the interaction. All benefits are given in the following Table S1, for all considered genotype-phenotype mappings and population stages.

Since in genotypes *G*_1_ $\equiv$ ([a],[a]) and *G*_2_ $\equiv$ ([a],[A]) there are 2 and 1 of the altruistic allele [a], resp., we obtain the following expression for the average benefit.

$$B\left( x \right):=2\left[ nb\frac{N}{2}x_{1}^{2}+\frac{n}{2}\left( 1 -\left( \frac{1-p}{2} \right)^{n-1} \right)bNx_{1}x_{2}+\frac{n}{4}\left( 1 -\left( \frac{3-2p}{4} \right)^{n-1} \right)b\frac{N}{2}x_{2}^{2} \right]+\frac{n}{2}\left( 1 -\left( \frac{1-p}{2} \right)^{n-1} \right)bNx_{1}x_{2}+n\left( 1 -\left( 1-p \right)^{n-1} \right)bNx_{1}x_{3}+\frac{n}{2}\left( 1 -\left( \frac{3-2p}{4} \right)^{n-1} \right)b\frac{N}{2}x_{2}^{2}+\frac{n}{2}\left( 1 -\left( \frac{2-p}{2} \right)^{n-1} \right)bNx_{2}x_{3}=Nnb\left[ x_{1}^{2}+\left( 1 -\left( \frac{1-p}{2} \right)^{n-1} \right)x_{1}x_{2}+\frac{1}{4}\left( 1 -\left( \frac{3-2p}{4} \right)^{n-1} \right)x_{2}^{2}+\frac{1}{2}\left( 1 -\left( \frac{1-p}{2} \right)^{n-1} \right)x_{1}x_{2}+\left( 1 -\left( 1-p \right)^{n-1} \right)x_{1}x_{3}+\frac{1}{4}\left( 1 -\left( \frac{3-2p}{4} \right)^{n-1} \right)x_{2}^{2}+\frac{1}{2}\left( 1 -\left( \frac{2-p}{2} \right)^{n-1} \right)x_{2}x_{3} \right]=Nnb\left[ x_{1}^{2}+\frac{1}{2}\left( 1 -\left( \frac{3-2p}{4} \right)^{n-1} \right)x_{2}^{2}+\frac{3}{2}\left( 1 -\left( \frac{1-p}{2} \right)^{n-1} \right)x_{1}x_{2}+\left( 1 -\left( 1-p \right)^{n-1} \right)x_{1}x_{3}+\frac{1}{2}\left( 1 -\left( \frac{2-p}{2} \right)^{n-1} \right)x_{2}x_{3} \right]$$

Secondly, let us calculate the average cost of the altruist gene from the interaction. All costs are given in the following Table S2, for all considered genotype-phenotype mappings and population stages.

Similarly, to what we did for the benefit function $B\left( x \right)$, for the average cost we have

$$C\left( x \right):=2\left[ nc\frac{N}{2}x_{1}^{2}+\frac{n}{1+p}\left( 1-\left( \frac{1-p}{2} \right)^{n-1} \right)cNx_{1}x_{2}+\frac{n}{1+2p}\left( 1-\left( \frac{3-2p}{4} \right)^{n-1} \right)c\frac{N}{2}x_{2}^{2} \right]+\frac{pn}{1+p}\left( 1-\left( \frac{1-p}{2} \right)^{n-1} \right)cNx_{1}x_{2}+n\left( 1 -\left( 1-p \right)^{n-1} \right)cNx_{1}x_{3}+\frac{2pn}{1+2p}\left( 1-\left( \frac{3-2p}{4} \right)^{n-1} \right)c\frac{N}{2}x_{2}^{2}+n\left( 1-\left( \frac{2-p}{2} \right)^{n-1} \right)cNx_{2}x_{3}=Nnc\left[ x_{1}^{2}+\frac{1+p}{1+2p}\left( 1-\left( \frac{3-2p}{4} \right)^{n-1} \right)x_{2}^{2}+\frac{2+p}{1+p}\left( 1-\left( \frac{1-p}{2} \right)^{n-1} \right)x_{1}x_{2}+\left( 1 -\left( 1-p \right)^{n-1} \right)x_{1}x_{3}+\left( 1-\left( \frac{2-p}{2} \right)^{n-1} \right)x_{2}x_{3} \right].$$

Comparing the benefit and the cost, the function $H\left( x \right)=B\left( x \right)-C\left( x \right)$ shows the total effect of the altruistic interaction for the altruistic gene [a].

For the sake of brevity, we introduce

$\alpha=1 -\left( \frac{1-p}{2} \right)^{n-1}, \beta=1 -\left( \frac{3-2p}{4} \right)^{n-1}$, $\gamma=1 -\left( 1-p \right)^{n-1}, \delta=1 -\left( \frac{2-p}{2} \right)^{n-1}$.

These quantities are positive for $p>0$. Then

$$H\left( x \right)=Nn\left[ \left( b-c \right)x_{1}^{2}+\beta\left( \frac{1}{2}b-\frac{1+p}{1+2p}c \right)x_{2}^{2}+\alpha\left( \frac{3}{2}b-\frac{2+p}{1+p}c \right)x_{1}x_{2}+ \gamma\left( b-c \right)x_{1}x_{3}+ \delta\left( \frac{1}{2}b-c \right)x_{2}x_{3} \right].$$

This quadratic form is positive (nonnegative) in the positive octant $x\geq0$, if all of its coefficients are nonnegative, i.e., the following conditions are met:

$$b\geq c, b\geq\frac{2+2p}{1+2p}c, b\geq\frac{4+2p}{3+3p}c, b\geq2c.$$

The most stringent bound is $b\geq2c$; it is sufficient to require this whenever $p>0$. It is also necessary, because if $x_{1}=0$ and $0{<x}_{2}\ll x_{3}$ (thus $x_{3}$ is very close to $1$, and $x_{2}$ is infinitesimally small), then the dominant term in $H\left( x \right)$ is the one containing $x_{2}x_{3}$. One can easily see that this is also the case for $p=0$, where $\gamma=\delta=0$, and

$$H\left( x \right)=Nn\left[ \left( b-c \right)x_{1}^{2}+\frac{7}{16}\left( \frac{1}{2}b-c \right)x_{2}^{2}+\frac{3}{4}\left( \frac{3}{2}b-2c \right)x_{1}x_{2} \right].$$

The sufficiency of the condition $b\geq2c$ is obvious, and the necessity also becomes transparent when $x_{1}=0<x_{2}$.

Table S-1. The altruistic benefit of altruism in different families.

| Genotypes of parents | Average number of couples | Average number of saved family members, for genotypes *G*_1_, *G*_2_, *G*_3_ | | |
| --- | --- | --- | --- | --- |
|  |  | *G*_1_ $\equiv$ ([a],[a])  altruistic | G_2_ $\equiv$ ([a],[A])  mixed phenotype $p$ | G_3_ $\equiv$ ([A],[A]),  selfish |
| *G*_1_$\times$*G*_1_ | $\frac{N}{2}x_{1}^{2}$ | $n_{1(11)}=nb$ | $n_{2(11)}=0$ |  |
| *G*_1_$\times$*G*_2_ | $Nx_{1}x_{2}$ | $\frac{n}{2}\left( 1 -\left( \frac{1-p}{2} \right)^{n-1} \right)b$ | $\frac{n}{2}\left( 1 -\left( \frac{1-p}{2} \right)^{n-1} \right)b$ |  |
| *G*_1_$\times$*G*_3_ | $Nx_{1}x_{3}$ | $n_{1(13)}=0$ | $n\left( 1 -\left( 1-p \right)^{n-1} \right)b$ |  |
| *G*_2_$\times$*G*_2_ | $\frac{N}{2}x_{2}^{2}$ | $\frac{n}{4}\left( 1 -\left( \frac{3-2p}{4} \right)^{n-1} \right)b$ | $\frac{n}{2}\left( 1 -\left( \frac{3-2p}{4} \right)^{n-1} \right)b$ |  |
| *G*_2_$\times$*G*_3_ | $Nx_{2}x_{3}$ | $n_{1(23)}=0$ | $\frac{n}{2}\left( 1 -\left( \frac{2-p}{2} \right)^{n-1} \right)b$ |  |
| *G*_3_$\times$*G*_3_ | $\frac{N}{2}x_{3}^{2}$ | $n_{1(33)}=0$ | $n_{2(33)}=0$ |  |

Table S-2. The altruistic cost of altruism in different families.

| Genotypes of parents | Average number of couples | Average number of saved family members, for genotypes *G*_1_, *G*_2_, *G*_3_ | | |
| --- | --- | --- | --- | --- |
|  |  | *G*_1_ $\equiv$ ([a],[a])  altruistic | G_2_ $\equiv$ ([a],[A])  mixed phenotype $p$ | G_3_ $\equiv$ ([A],[A])  selfish |
| *G*_1_$\times$*G*_1_ | $\frac{N}{2}x_{1}^{2}$ | $n_{1(11)}=-nc$ | $n_{2(11)}=0$ |  |
| *G*_1_$\times$*G*_2_ | $Nx_{1}x_{2}$ | $-\frac{n}{1+p}\left( 1-\left( \frac{1-p}{2} \right)^{n-1} \right)c$ | $-\frac{pn}{1+p}\left( 1-\left( \frac{1-p}{2} \right)^{n-1} \right)c$ |  |
| *G*_1_$\times$*G*_3_ | $Nx_{1}x_{3}$ | $n_{1(13)}=0$ | $-n\left( 1 -\left( 1-p \right)^{n-1} \right)c$ |  |
| *G*_2_$\times$*G*_2_ | $\frac{N}{2}x_{2}^{2}$ | $-\frac{n}{1+2p}\left( 1-\left( \frac{3-2p}{4} \right)^{n-1} \right)c$ | $-\frac{2pn}{1+2p}\left( 1-\left( \frac{3-2p}{4} \right)^{n-1} \right)c$ |  |
| *G*_2_$\times$*G*_3_ | $Nx_{2}x_{3}$ | $n_{1(23)}=0$ | $-n\left( 1-\left( \frac{2-p}{2} \right)^{n-1} \right)c$ |  |
| *G*_3_$\times$*G*_3_ | $\frac{N}{2}x_{3}^{2}$ | $n_{1(33)}=0$ | $n_{2(33)}=0$ |  |

**SI: References**

1. Garay J, Garay BM, Varga Z, Csiszár V, Móri TF. To save or not to save your family member’s life? Evolutionary stability of self-sacrificing life history strategy in monogamous sexual populations. BMC Evol. Biol. 2019;19:147.
